# Human Stiffness Perception and Learning in Interacting With Compliant Environments

**DOI:** 10.3389/fnins.2022.841901

**Published:** 2022-06-06

**Authors:** Chie Takahashi, Morteza Azad, Vijaykumar Rajasekaran, Jan Babič, Michael Mistry

**Affiliations:** ^1^School of Computer Science, University of Birmingham, Birmingham, United Kingdom; ^2^Edinburgh Centre for Robotics, School of Informatics, University of Edinburgh, Edinburgh, United Kingdom; ^3^School of Metallurgy and Materials, University of Birmingham, Birmingham, United Kingdom; ^4^Department of Psychology, University of Cambridge, Cambridge, United Kingdom; ^5^Laboratory for Neuromechanics and Biorobotics, Department for Automation, Biocybernetics and Robotics, Jožef Stefan Institute, Ljubljana, Slovenia; ^6^Faculty of Electrical Engineering, University of Ljubljana, Ljubljana, Slovenia

**Keywords:** stiffness perception, force perception, visuomotor control, learning, sensorimotor prediction, perceptual decision

## Abstract

Humans are capable of adjusting their posture stably when interacting with a compliant surface. Their whole-body motion can be modulated in order to respond to the environment and reach to a stable state. In perceiving an uncertain external force, humans repetitively push it and learn how to produce a stable state. Research in human motor control has led to the hypothesis that the central nervous system integrates an internal model with sensory feedback in order to generate accurate movements. However, how the brain understands external force through exploration movements, and how humans accurately estimate a force from their experience of the force, is yet to be fully understood. To address these questions, we tested human behaviour in different stiffness profiles even though the force at the goal was the same. We generated one linear and two non-linear stiffness profiles, which required the same force at the target but different forces half-way to the target; we then measured the differences in the learning performance at the target and the differences in perception at the half-way point. Human subjects learned the stiffness profile through repetitive movements in reaching the target, and then indicated their estimation of half of the target value (position and force separately). This experimental design enabled us to probe how perception of the force experienced in different profiles affects the participants’ estimations. We observed that the early parts of the learning curves were different for the three stiffness profiles. Secondly, the position estimates were accurate independent of the stiffness profile. The estimation in position was most likely influenced by the external environment rather than the profile itself. Interestingly, although visual information about the target had a large influence, we observed significant differences in accuracy of force estimation according to the stiffness profile.

## Introduction

Humans can control their body movements to maintain balance when interacting with external forces, even in an uncertain environment. Imagine a situation when you are sitting down on a gym ball to perform an exercise. Before taking actual action, you may push the ball by hand several times to check its elastic stiffness. Such natural behaviour prepares us for forthcoming exercise on the soft gym ball. The Central Nervous System (CNS) learns an active contact motion through sensorimotor feedback and plastically adapts body movements to a new environment. Humans have remarkable capabilities to generalise sensory information in a cognitively robust way and optimise their control performance (Wolpert et al., 1995, 2011). Research on motor control suggests that humans develop internal models that allow them to formulate motor behaviour, to predict consequences of their action and to achieve the behavioural goal optimally (Davidson and Wolpert, 2003; Flanagan et al., 2003). Through exploring human reaching movements against applied force fields, several models have been proposed to explain the underlying mechanisms (Franklin et al., 2003; Krakauer et al., 2006; Chib et al., 2009; Darainy et al., 2009; Kadiallah et al., 2012; Judkins and Scheidt, 2014; Casadio et al., 2015). Online sensorimotor feedback and the learning process are key components in optimising human reaching behaviour. However, to our knowledge, how the brain interprets force perception and how the brain employs the perception to make an appropriate prediction are still open questions.

Using psychophysical measures such as discrimination thresholds, human perception of force has been investigated, mainly in terms of the magnitude and direction of the force (Jones and Tan, 2013; van Beek et al., 2013, 2015). A considerable amount of research has shown that human force perception is formed by haptic information experienced during touch, but it can also be influenced by other sensory information, predominantly visual (White, 2012; Li et al., 2015) and proprioceptive (van Beek et al., 2015). For example, the perceived heaviness of an object is subjectively changed by the “visual” object size; this is well known as the size-weight illusion (Murray et al., 1999; Ernst, 2009). In the case of the perception of force magnitude, previous studies (van Beek et al., 2013, 2015) reported anisotropic characteristics in both 2D and 3D, suggesting that the perception was affected by the direction of the postural arm movement. In real-world interactions with compliant surfaces, force profiles are varied and complicated; that is, when making a contact with an object and handling it, the profiles are governed by not a simple linear equation, rather by multiple or dynamic formulae. Humans examine material stiffness through active exploration, such as pushing the surface, and then can thereby anticipate the static stiffness. Although there are a few studies investigating human haptic perception using linear and nonlinear materials (Hartman et al., 2016; Wu and Klatzky, 2018; Guang et al., 2019), it is still unclear whether the brain understands the profile itself or not. Understanding the brain mechanism is important for designing systems optimally in human-machine interaction, for example, in virtual reality rehabilitation (Levac et al., 2019), robot-assisted rehabilitation (Gasperina et al., 2021), and human-robot collaborative tasks (Mauri et al., 2019; Roveda et al., 2019).

In terms of learning force profiles, motor control research has helped in understanding human goal-directed movements against external force fields (Bhushan and Shadmehr, 1999; Thoroughman and Shadmehr, 2000). Human sensorimotor performances have been studied by both empirical and computational approaches (Wolpert et al., 2011, for a review). Interacting with a force exerted by contact surfaces, humans can learn how to control their reaching movements optimally and how to adjust their arm impedance characteristics appropriately. Previous studies, employing an error-based visuomotor perturbation paradigm, have shown that a certain exposure (e.g., repetitive movements against compliant forces) facilitates learning spatial and temporal characteristics of the interaction (Goodbody and Wolpert, 1998; Krakauer et al., 2006). Trial-based learning models have also been studied to understand the underlying principles of human motor control (Krakauer et al., 2006). In a similar study employing a dynamic perturbation (Wang et al., 2001), the internal model seemed to be modified, integrating with sensory feedback through the learning process. The brain possibly controls complex movements by flexibly combining motor primitives, or elements, in the sensorimotor maps and transforming desired joint trajectories into motor commands via learning (Thoroughman and Shadmehr, 2000). Although learning can improve the motion performance, it is less known how the perception would be influenced by force profiles.

**FIGURE 1:**
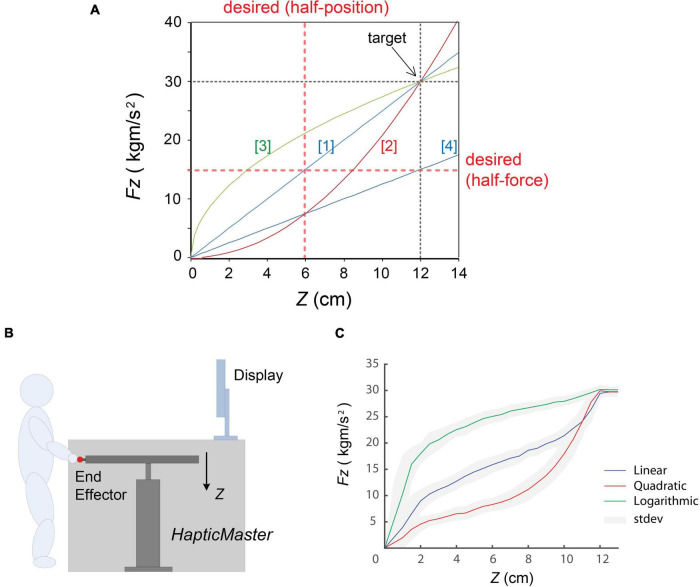
Experimental settings. **(A)** Four different types of force dynamics employed: (1) linear, (2) quadratic, (3) logarithmic, and (4) half-linear. Three stiffness profiles [(1), (2), and (3)] were designed to produce the same force 30 kg⋅m/s^2^ at *Z* = 12 cm, and (4) was the half of the (1), that is 15 kg⋅m/s^2^ at *Z* = 12 cm. The red dotted lines indicate the desired values in half-position and half-force prediction task. **(B)** A cartoon of the explanation of the experimental settings. **(C)** A typical example of motion profile of one participant in three stiffness conditions. Three coloured solid lines indicate averaged performance of force (*F*_*z*_) vs. position (*Z*). blue, linear model; red, quadratic model; green, logarithmic model; respectively. The grey areas represent standard deviations of the performance in each model. (In this figure, “noisy” trajectories during holding the end-effector around the target (at Z = 12 cm) were excluded).

The objectives of the current study were to explore the following research questions: (1) Do humans learn different environmental stiffness profiles while moving in contact with the environment? and (2) Do humans perceptually distinguish stiffness profiles? We hypothesised that if the brain could properly understand stiffness profiles through learning and if then the CNS could establish an internal model modulating movement properties in response to applied force, humans could appropriately perform reaching movements despite the perturbations caused by the stiffness of the environment. To systematically explore our hypotheses, we employed an experimental paradigm where the subjects had to perform arm-movements in contact with environment that had three different stiffness profiles. We designed the three stiffness profiles such that they had a single intersection exactly at the point that was defined as the movement target (see Figure 1A). To quantify the reaching performance and the learnability, we measured the position of the hand and the force of the interaction throughout the motion. To acquire the understanding of the stiffness profiles, we also asked the subjects to move the hand to and hold it at certain points before the target where the three stiffness profiles were significantly different. Analysing the hand position and the force with respect to the stiffness profiles enabled us to get insight into how the brain interprets different environmental stiffness.

## Experiment 1

### Materials and Methods

#### Participants

A total of 36 subjects (28 females, 18–41 years old, mean age: 20.0 ± 3.8 (SD), mean height: 169.1 ± 6.4 cm (SD), 32 right-handed) took part in the main experiment. All the participants had normal or corrected to normal vision, and no known motor deficits and/or any limb injuries. The study was approved by the Research Ethics Committee, University of Birmingham, and all procedures were in accordance with the Declaration of Helsinki. Participants were recruited via the University Research Participant Scheme—SONA System—and they received £10 for their participation. The subjects gave informed consent to participate but were naive to the purpose of the experiment. They also had no prior knowledge about the stiffness models.

#### Apparatus and Stimuli

The study was conducted using a 3-degrees-of-freedom (3-DoF) haptic device, HapticMaster (Moog Inc.), which consisted of a large robotic manipulator with an end-effector (van der Linde and Lammertse, 2003). A simple spring force (*F*) was generated by setting parameters (i.e., spring stiffness, *k*) in real-time depending on the end-effector position (*Z*). We designed four different compliant forces by changing the stiffness *k* values (see Figure 1A and Table 1). The *k* value is maintained constant in two linear models ([1], [4]) and position dependent in other two non-linear models ([2], [3]). Three stiffness profiles were set crossing at the same position (*Z* = 12 cm) and the same force (*Fz* = 30 kg⋅m/s^2^) as a “target.”

**TABLE 1:** Spring type, stiffness and force.

	Type	Stiffness	Force
Linear	Full [1]	250	250 Z
	Half [4]	125	125 Z
Non-Linear	Quadratic [2]	2083 Z	2083 Z^2^
	Logarithmic [3]	86.6 Z^–1/2^	86.6 Z^1/2^

*The four different forces are rendered by changing the spring stiffness.*

A participant stood in front of the haptic device and grasped the end-effector (Figure 1B). The apparatus was fully covered by black cloth, so he/she could not see his/her hand and any movements of the rod. The initial, or “home,” position, where the centre of the end-effector was *Z* = 0 at the workspace, was 110 cm above from the ground. When the applied force was zero, the end effector was stably situated at the home position as an end of a spring. In this study, the rod movements were restricted along the x and y direction. This ensured the participant’s movements or the exerted force at the end point was only along the z direction.

The visual information about the task such as the target positions was provided by the 21-inch flat display which was approximately 1.60 m away from the participants’ standpoint. The centre of the screen was aligned to the centre of the robotic rod and approximately 1.55 m above the ground (Figure 1B). In order to remove external disturbances and any predictive cues such as noises caused by rod movements, participants listened to alpha-wave music through headphones during the task.

#### Experiment Protocol

**FIGURE 2:**
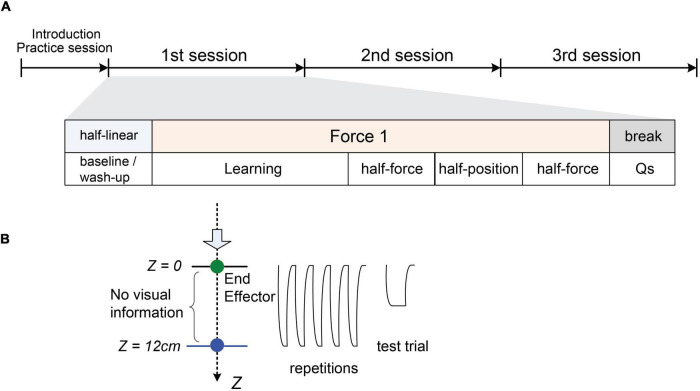
Experimental protocol. **(A)** A diagram of the four sessions. Following the practice, each session consisted of a series of five blocks: (1) baseline/wash-up, (2) learning, (3) half-force estimation task, (4) half-position estimation task, (5) half-force estimation task, and then questionnaire periods. The baseline/wash-up employed the half-linear condition. Then, one of three types of stiffness profiles (linear, quadratic, logarithmic) was assigned pseudo-randomly after the baseline/wash-up. **(B)** A cartoon illustrating the repetitive movements to reach the target (*Z* = 12 cm, *Fz* = 30 kg⋅m/s^2^) and a test trial.

The experiment consisted of four sessions (see Figure 2A) with the four different stiffness profiles described above and in Figure 1A. We probed the performance at the target and at half of either position or force in the course of the stiffness profile. The profile [4] was for practice and set as a reference of desired half-force. Firstly, a practice session was introduced using only the “half-linear” to learn the task. After the practice, one stiffness from three different profiles was *pseudo-randomly* assigned to the session orders. Each session comprised a series of blocks: (1) “baseline/wash-up,” (2) learning, (3) “half-force” estimation task, (4) “half-position” estimation task, and then (5) “half-force” estimation task. In each block, participants were asked to give their estimations on test trials after a certain number of repetitive reaching movements to the target, or reference (Figure 2B). The test trials measured how accurately participants held the end-effector compared to the desired physical property and probed whether they had properly perceived the different stiffness profile to estimate it.

Each session started from the “baseline” block set with the “half-linear” condition aiming to reset, or “wash up,” the motor learning and motor memory. This block consisted of five sets of (three repetitive movements followed by one test trial). After the baseline/wash-up, the stiffness was assigned to one of the three stiffness profiles. The “learning” block consisted of 10 sets of a consecutive task (five repetitive movements with feedback followed by one test trial). On the test trial in both “baseline/wash-up” and “learning” blocks, participants held the end-effector at the *same* force that they had experienced at the target in the repetitive period. Following the “learning” block, participants conducted three test blocks. These blocks consisted of five sets, the same as the “baseline/wash-up,” In the “half-position” test block, participants held the end-effector at the position where they estimated as the half of the real target position that they learnt through the repetitive movements prior to the trial. In the “half-force” test block, they held the end-effector with half the force they estimated from the previously experienced force at the target. We conducted a pilot experiment in advance, and we observed that the “half-force” prediction performance was worse than “half-position” performance. Thus, in this experiment we doubled the number of “half-force” trials, aiming to keep the standard deviation of “half-force” prediction performance being not so far from the standard deviation of “half-position” one; one “half-position” block was set between two “half-force” blocks.

In preliminary testing the obtained data suggested that participants might have paid attention to timing rather than to stiffness during the repetitive movements in the learning session. If there were no time constraint, participants might maintain their own rhythms to reach the anticipated goal. Such timing moreover is closely related to speed control and so could affect the force itself. Several studies have shown that time perception plays an important role in human motor control (Goodbody and Wolpert, 1998; Rank and Di Luca, 2015; Berret and Jean, 2016). Thus, considering these points we designed a certain time window for the reaching movements, with visual feedback to the participants. The average time window was calculated on the basis of preliminary testing and was held the same for the different stiffness conditions. Visual information was provided only at the beginning and end of the movement, in view of its effect on human force perception.

#### Experimental Procedure

The total of 36 participants were equally divided into six groups with six order combinations, in order to minimise the order effects on the performance. Prior to the experiment, all participants received instructions about the task from the experimenters and then began the practice session followed by the three main sessions. Participants moved an end-effector of a robotic manipulandum against force generated by haptic devices following the experimental protocol. They pushed the end-effector to reach the target, and then released it to freely return to the initial position (*Z* = 0). The end-effector movements were monitored by the computer systems and visual information was provided in real-time on the screen, excluding a certain area between the start position and the target position. Participants conducted repetitive movements against the force to learn the reference force produced by the stiffness profile and then made a prediction based on it. In the repetitive period, the target zone was visually defined by a blue coloured circle. Participants were asked to reach the target within a certain time window (0.6 ± 0.2 s). The timer started when the end-effector moved from the initial position and stopped when the end effector position crossed the target position. Participants received a feedback message for each movement on the display: “too fast” when <0.4 s or “good timing” 0.4 s ≤ *t* ≤ 0.8 s, or “too slow” when *t* > 0.8 s. Depending on the timing of the reach, the target colour correspondingly changed from blue into either yellow or light blue, or red. They were also requested to set the end-effector within the target zone (which was displayed as a small circle) as accurately as possible and to keep it there for 1 s, maintaining it with zero-velocity as much as possible. In the testing period, no visual feedback was provided. Participants kept the end-effector indicating their prediction, and maintained it with zero-velocity for 3 s. On test trials in the “practice” and the “learning” blocks, participants kept the end-effector with the same feeling as for the target force. In the “half-force” or “half-position” blocks, they held the end-effector (1) at half the position they were experiencing with the target or (2) at half the force they were experiencing with the target. After the completion of each session, the participant had a break and was asked to answer a few questions aiming to assess subjective perception of the force he/she experienced. The total experimental time for each participant was approximately 90 min.

The physical properties in the movements were measured with sensors mounted at the end-effector while the human subjects were controlling the haptic device in different force conditions. The end-effector’s position *Z*, the velocity *Vz*, and the force *F* were recorded at a 5-ms sampling rate. We analysed the movement performance, and participants’ predictions on the test trials.

#### Learning Analyses

In the learning block, participants moved the end-effector toward the target and held it at the target as accurately as possible for 1 s with zero velocity. Aiming to examine how the reaching performance was changing through the repetitive movements in three different stiffness conditions, we introduced a metric to evaluate the position accuracy:


(1) $$\text{M}=\left|\left(Z-Z_{d}\right)\right|+K*\left|\left(\dot{Z}-{\dot{Z}}_{d}\right)\right|$$

where, Z is the end-effector position reached at the end of each movement and $\dot{Z}$ is the velocity maintained there; *Z_d* and ${\dot{Z}}_{d}$ are the desired position (12 cm) and the desired velocity (0.0 m/s), respectively. *K* is maintained as a constant value (0.005 s) defined by the sampling rate employed in the experimental setting. Thus, the metric (M) indicates the position accuracy in reaching the target; the unit of M is cm. In other words, M = 0 means that a participant had reached the target accurately and maintained the velocity at zero. (Note that this learning analysis excluded the trial data performed without visual feedback.).

#### Data Analysis

The arm manipulator device provided position, velocity, and force information, which were used to compute the learning metric M and to analyse force perception. The position and velocity information were also captured when the participant reached the target zone, identified by a specific identity variable defined in the software. The mean learning metric value was computed for each participant over the set of 50 repeated trials in different stiffness profiles. A Repeated Measures ANOVA and paired *t*-tests were performed (using IBM SPSS software) to evaluate the participant’s individual learning and perception performance in “linear,” “quadratic,” and “logarithmic” profiles.

### Results

#### Reaching Performances in Different Stiffness Profiles

Figure 1C shows an example of the typical motion profile of one participant through 50 repetitive movements with visual feedback in the learning block, where the target was located at *Z* = 12 cm with *Fz* = 30 kg⋅m/s^2^. (This figure shows the analysed reaching performances excluding the noisy holding period). These trajectories clearly indicate how the real movement properties, i.e., *Fz* vs. *Z*, were different in the three stiffness models, which generated “linear,” “quadratic,” and “logarithmic” force profiles.

**FIGURE 3:**
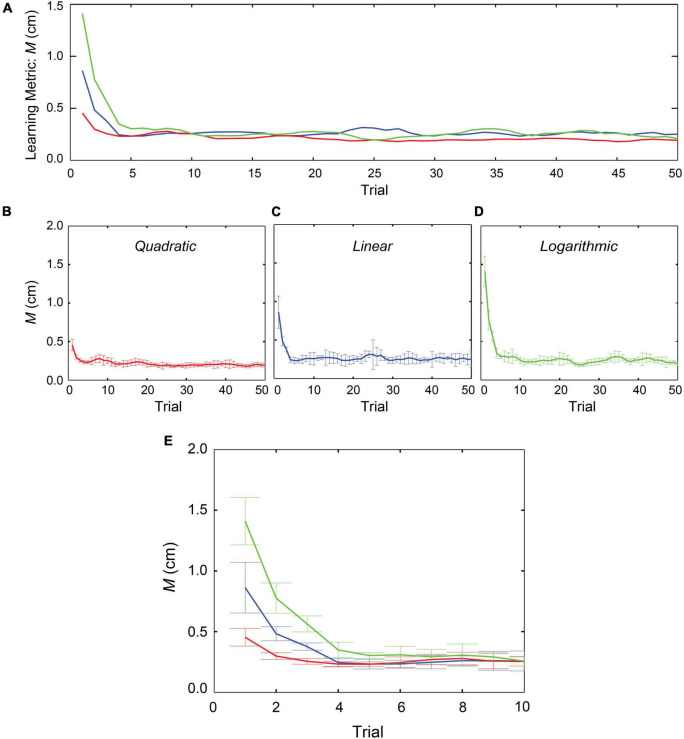
Changes in learning metrics in three conditions. Evaluation of 36 participants over the course of the 50 repetitions. The graphs represent the means with standard errors of the M values in three different stiffness profiles. **(A)** Learning-curves in all the three stiffness models. **(B)** Learning performance in “quadratic” model. **(C)** In “linear” model. **(D)** In “logarithmic model. **(E)** Focusing on the initial 10 trials.

Figure 3 shows changes in learning metric (M) value over the 50 repetitions in “linear,” “quadratic,” and “logarithmic” profiles; the data were averaged across 36 participants. Three coloured lines all indicate that the averaged M values changed by more than two orders of magnitudes within the initial 10 repetitions in the learning block and then gradually improved in accuracy over the course of the block. Although none of the learning metrics ever converged to zero, they were all changing in a direction indicating improvements in accuracy. Inspection of the three learning-curves in Figure 3E suggests that the curve for the “quadratic” case is more accurate than those for the other two cases. The standard errors for position accuracy also indicated that the performance in the quadratic case was more reliable in reaching the target position.

We calculated each participant’s metric (M) value averaging across the 50 repetitions. A repeated measures ANOVA with a Greenhouse-Geisser correction showed that mean M values did not differ significantly between the three stiffness conditions [*F*(1.228, 42.963) = 2.802, *p* = 0.094]. This result suggests that when humans performed repetitive movements to reach the target, the accuracies were maintained at the end goal position irrespective of the different stiffness profiles.

From Figure 3E, we observed that a major change in the learning performance occurred within the initial 10 trials. We calculated each participant’s M value averaging across the 10 repetitions and conducted statistical analyses using a repeated measures ANOVA for the three different forces. The results with Greenhouse-Geisser corrections showed that there were significant differences in the learning metrics among the stiffness profiles [*F*(1.605, 56.188) = 7.209, *p* = 0.003]. *Post hoc* tests using Bonferroni correction showed that between the “linear” case (*M* = 3.2E-3, *SD* = 2.2E-3) and the “quadratic” case (*M* = 2.6E-3, *SD* = 1.26E-3) there was no significant difference [*t*(35) = 1.300, *p* = 0.202]. In contrast, between the “linear” and the “logarithmic” cases (*M* = 4.3E-3, *SD* = 2.41E-3) there was a marginal statistical difference [*t*(35) = -2.101, *p* = 0.0043], and between “quadratic” and the “logarithmic” cases there was a significant difference [*t*(35) = -4.307, *p* = 0.000]. Despite showing high standard deviations, these results suggest that the performance was significantly affected by the stiffness profile in the initial stages of the learning phase.

#### Stiffness Perception in Different Force

**FIGURE 4:**
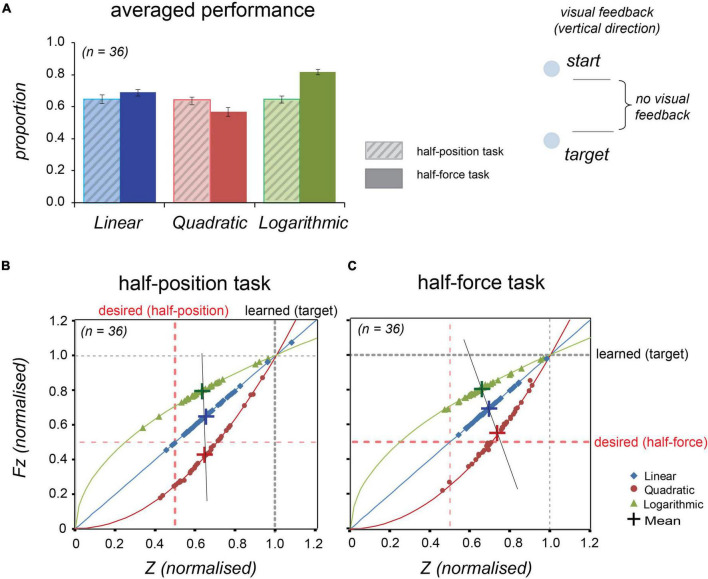
Stiffness perception in three force dynamics in Experiment 1. Thirty-six participants’ performance in the “half-position” and “half-force” test trials. Blue, red, and green colours, respectively, represent linear, quadratic, and logarithmic stiffness conditions. **(A)** Normalised averaged performance in the half-position trials (light-coloured bars with diagonal lines, on the left sides) and in the half-force trials (dark-coloured bars, on the right sides). Error bars denote ± 1 standard error. **(B)** In the half-position trials, the individual data were plotted onto the lines representing the stiffness dynamics. **(C)** In the half-force trials. The individual data averaged in the test trials were plotted onto the line representing the force dynamics. Large crosses represent the mean values and black line is regression line fitted to the shown means.

The read-out data (position and force) at the end of the trials in both testing blocks were analysed and statistically evaluated. To allow for individual differences in absolute performance, all participants’ data were averaged over the total trials in the test block and then normalised by their average performance on the test trials in the learning block; thus, the proportion (0.5) is the desirable value in each condition. See Figure 4A and Table 2. The individual data (*n* = 36) were plotted on to the lines representing the force; Figure 4B shows the half-position task performance and Figure 4C shows the half-force task performance. These graphs indicate that most participants overestimated the real half-position (at 0.5 as *Z* = 6 cm) in all three forces; also, the majority of participants overestimated the actual half-force (at 0.5 as *Fz* = 15 kg⋅m/s^2^).

**TABLE 2:** Summary of comparison between the half-position task performance vs. half-force task performance at the test trials in Experiment 1.

	z-Position				z-Force			
Force types	Half-position task		Half-force task		Half-position task		Half-force task	
	Mean	*SD*	Mean	*SD*	Mean	*SD*	Mean	*SD*
Linear	0.653	0.113	0.704	0.096	0.662	0.125	0.713	0.107
Quadratic	0.645	0.121	0.744	0.106	0.432	0.162	0.569	0.159
logarithmic	0.633	0.112	0.666	0.113	0.802	0.078	0.821	0.080

*Mean, averaged across 36 participants (5 trials for the half-position, 10 trials for the half-force); SD, Standard deviation.*

Figure 4A shows that there were no significant differences on half-position estimations among the three different types of stiffness. A repeated measures ANOVA shows that there were no significant differences on half-position estimations in three different force profiles: *F*(2, 70) = 0.627, *p* = 0.537. These results indicate that the participants estimated the similar position regardless of the force. In contrast to the half-position task, there were significant differences on half-force estimations among three different force profiles. A repeated measures ANOVA with a Greenhouse-Geisser corrections shows that there were significant differences in half-force estimations in three stiffness conditions: *F*(1.175, 50.944) = 105.443, *p* = 0.000. These results suggest that the half-force estimations were affected by the stiffness profiles.

In further statistical analyses, a paired-samples *t*-test was conducted to compare the estimation performance between the half-position and half-force tasks. There were significant differences in positions measured at half estimation between the two tasks, *t*(35) = 2.27, *p* = 0.029 in “linear” force and *t*(35) = 4.74, *p* = 0.000 in “quadratic” force, suggesting that the participants understood the task and learned the force. Conversely, there were not significant task differences in half-estimation in “logarithmic” force, *t*(35) = 1.66, *p* = 0.106, These trends were also shown in forces measured at half-estimation in the two tasks, *t*(35) = 2.31, *p* = 0.027 in “linear” force and *t*(35) = 4.58, *p* = 0.000 in “quadratic” force; in contrast there was not a significant task difference in measured forces in “logarithmic” case, *t*(35) = 1.62, *p* = 0.114. Such results suggest that the participants did not well distinguish the task itself, or they could not estimate the half, in “logarithmic” force profile.

In graphical examinations of the scatter plots, the mean values of half-position estimations were equally shifted from the desired value (red-dotted line) in three different stiffness conditions. This trend indicates that participants overestimated the half-position equally regardless of the force. In contrast, a black straight line connecting respective mean values of half-force estimations was tilted from the desired red-dotted line, suggesting that the performance was affected by the stiffness profile.

## Experiment 2

Based on the results in Experiment 1, we conducted additional testing to examine the effect of direction of visual feedback on estimations. In Experiment 1, the movement direction of the displayed circle representing the end-effector position was vertical and the same as the actual physical movements. Although all the visual information disappeared in the estimation trials, participants might have learned, by means of proprioception, the relative position of their body, rather than the force profile itself. To examine this, we designed an experiment to provide the visual feedback independently of the direction of hand movement.

### Materials and Methods

#### Participants

Fourteen participants [4 female, 22–31 years old, mean age: 25.1 ± 2.3 (SD)] were newly recruited. The recruitment procedures and the criteria were identical to those of Experiment 1. Experiment 2 was treated as a follow-up to the Experiment 1, so that the number of participants was relatively small.

#### Stimuli and Procedure

**FIGURE 5:**
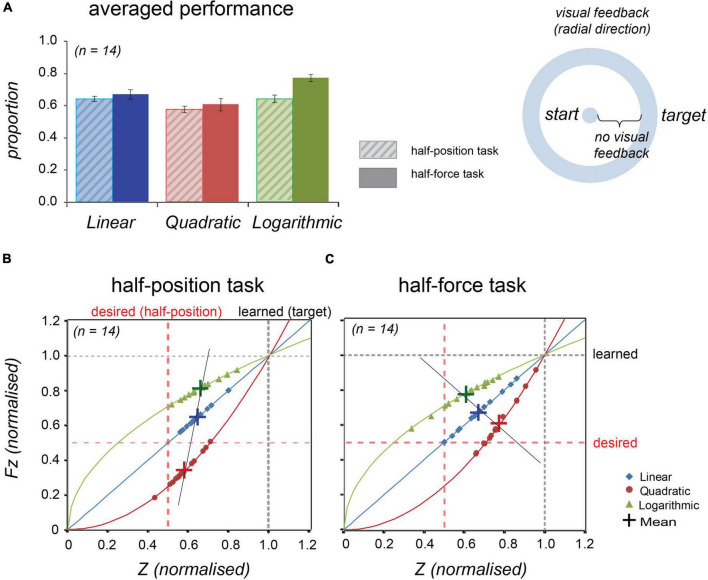
Averaged normalised performance across 14 participants at the half-position and at the half-force trials in Experiment 2. Blue, red, and green colours, respectively, represent linear, quadratic, and logarithmic stiffness conditions. **(A)** Normalised averaged performance in the half-position trials (light-coloured bars with diagonal lines, left) and in the half-force trials (dark-coloured bars, right). Error bars denote ± 1 standard error. **(B)** In the half-position trials, the individual data were plotted onto the lines representing the stiffness dynamics. **(C)** In the half-force trials. The individual data averaged in the test trials were plotted onto the line representing the force dynamics. Large crosses represent the mean values and black line is regression line fitted to the shown means.

The task information was displayed on a screen and guided a participant to move an end-effector to reach the target. The initial position was visually indicated as the centre of a circle on the screen and the target was set at the contour. The visual information was synchronised with the end-effector movements in the same manner with the Experiment 1, but its direction was radial, not vertical (see the cartoon in Figure 5 and compare with the Figure 4).

The experimental procedure and the protocol were broadly similar to the main experiment: three sessions with different stiffness conditions started from the “wash-up/baseline,” followed by the “learning,” “half-force,” and “half-position” blocks. The learning block consisted of 5 sets of a task (5 repetitions with feedback followed by one test trial), and the “half-force” block was only once per each session. The total experimental time was approximately 60 min and shorter than the Experiment 1. After Experiment 1, we noticed that the experimental duration (90 min) was quite long and caused participants’ tiredness; thus, we shortened it aiming to keep their concentration.

### Results

#### Effects of Visual Feedback Direction

As shown in Figure 5A and Table 3, the averaged half-estimation performances in Experiment 2 had a similar pattern to those of Experiment 1. The individual data (*n* = 14) were also plotted on to the solid lines representing the force profile (see Figures 5B,C). A repeated measures ANOVA was conducted for the half-estimation values in three stiffness conditions; the estimations differed significantly among forces in both tasks: *F*(2, 26) = 7.241, *p* = 0.003 in the half-position and *F*(2, 26) = 7.948, *p* = 0.002 in the half-force.

**TABLE 3:** Summary of comparison between the half-position task performance vs. half-force task performance at the test trials in Experiment 2.

	z-Position				z-Force			
Force types	Half-position task		Half-force task		Half-position task		Half-force task	
	Mean	*SD*	Mean	*SD*	Mean	*SD*	Mean	*SD*
Linear	0.643	0.065	0.670	0.106	0.643	0.065	0.670	0.107
Quadratic	0.576	0.076	0.772	0.091	0.340	0.089	0.607	0.145
logarithmic	0.656	0.090	0.608	0.124	0.807	0.055	0.773	0.084

*Mean, averaged across 14 participants (5 trials for the half-position, 10 trials for the half-force); SD, Standard deviation.*

Inspection of the data suggests that the half estimation performances in all the three conditions show similar trends in Experiment 1 and Experiment 2. Noticeably the slope of a line connecting respective mean values of the half-position estimations was more tilted from the desired dotted line, showing that performance was slightly worse than that of Experiment 1. Conversely, the line slope of the half-force estimations approached the desired dotted line, showing that the performance was slightly improved. These changes suggested that performance was likely to have been affected by the direction of visual feedback.

## Discussion

This study investigated human perception of force and the learning performance by systematically manipulating stiffness profiles. We generated three different position-force profiles having one intersection as a target. Participants moved the end-effector of the haptic device to the target in the different conditions and indicated their estimation of half of the target value in either position or force. Firstly, although we observed statistically significant differences in the early stages of motor learning, the differences in position accuracies between the profiles diminished through the repetitive movements. Secondly, our results showed better estimations in the half position condition than in the half force regardless of the force profiles. Importantly, there were significant differences in the half-force estimations depending on the profile, suggesting that the CNS can differentiate it. The findings demonstrated that, even though they were less accurate, human estimations of stiffness were certainly affected by the profile through active learning. However, there were several other factors (e.g., time perception, velocity control in repetitive movements), that might have led to inaccurate performances such as over-estimations in reaching the anticipated target. Moreover, the results also indicated difficulties in one specific force profile, here the logarithmic case, in both learning and prediction performances, suggesting that these might have been influenced by a degree of familiarity in force profiles in everyday situations. Such possible factors closely interact with each other; thus, these should be carefully discussed to understand the mechanisms.

### Force and Motor Learning in Different Stiffness Profiles

The learning analysis evaluated changes in the position accuracies through repetitive movements under three different stiffness conditions. The results showed that although none of the metric (M) values ever converge to zero, the participants were gradually improving the accuracy within a minor range of deviation. Despite the learning duration and although the analyses were different from those of other studies, our data showed similar learning trends to others; for example, the mirror-tracing task in Stratton et al. (2007) study, where the individual learning curves were characterised by fast time scales in the “warm-up” period and slow scales in the “persistent change” period.

The learning metric for all participants showed that the overall target reaching performance was not affected by the different stiffness profiles. All were able to reach the target position with better accuracy over the course of the trials. Among three different types of stiffness in the current study (Figure 3A), the learning performance was relatively better in the case of the “quadratic” force profile and the improvements were progressed through the repetitive movements. In the initial stages of the learning phase (Figure 3E), the participants showed reaching performance with high deviations in all the three forces, but the deviations were reduced, even in the case of “logarithmic” force, within a few repetitions. However, the learning-curve in the “quadratic” force condition was observed to be consistent throughout the trial, suggesting that participants might have easily understood this type of force over the course of repetitions. In contrast, the learning metric value in the “logarithmic” force was slightly worse in comparison with other two conditions. Therefore, the participants might have had needed more repetitions to understand the logarithmic case and improve their accuracy in reaching the target position.

Inspecting the results from a different perspective, human perception of force is very subjective, so the results inevitably depend on each participant’s sensitivity; thus, we need to consider interactions with other cognitive functions. Previous studies have shown that time perception significantly affects the human perception of force; for example, Rank and Di Luca (2014) demonstrated that the arm movement performance changed with a time-dependent force perception. They found that the response time and the arm movement influenced the accuracy. Thus, a speed control in reaching movements is a key factor that would also affect force perception. We originally designed the experiments with no time-limitation to reach the target, aiming to provide participants enough time to understand the different force profiles. However, in a pilot experiment, we noticed that participants tended to generate their own rhythms and maintain them in repetitive reaching movements, regardless of the stiffness profiles. Such rhythmic, or speed, control might have led to less accuracy in reaching the target and induced scant attention to the profiles.

In this study, although timing feedback was roughly provided to the participants when the end-effector was reaching at the target, there was no strict time limitation. We observed that the averaged reaching times were slightly different in the three force conditions: “linear”: 0.624 s ± 0.049 and “logarithmic”: 0.652 s ± 0.036) were shorter than the quadratic case (0.681 s ± 0.053). As can be seen in Figure 1A in “Materials and Methods” section, the magnitudes of linear and logarithmic forces were more than double those for the quadratic case at the short distance. Consequently, large stiffness at the early movements might have caused difficulties in controlling the timing when generating force against the spring stiffness. Because the participants tended to approach the target more rapidly in linear and logarithmic force trials, this might have impaired judgements and the accurate learning of the profile. Such differences in reaching time inevitably depend on the profile, and this might have affected the learning performance and the force perception. A potential enhancement of our study would be to record differences in body posture and in electromyographic signals during the trials. These measures could then be related to variation in motor timing and rhythms according to the stiffness profiles.

By interviews and questionnaires at the end of each session, we also examined subjective interpretation of the different stiffnesses (see Supplementary Material 3). In Experiment 2 where we introduced visual feedback in a radial direction, one third of participants admitted that the “quadratic” force profile resembled pushing a cushion, and half of participants claimed/reported that the “logarithmic” force profile resembled pushing a revolving door. The “quadratic” case might have evoked a mental image of pushing common materials in daily experience, whereas participants might have been unfamiliar with the logarithmic one. Although these were very rough examinations, such exploration could be supported by other research in relation to motor learning and episodic or procedural motor memory (Dayan and Cohen, 2011). To investigate familiarity effects, additional experiments would be considered, for example, by manipulating visual image to associate with different stiffness profiles.

### Stiffness Perception and Other Factors in Motor Control

A considerable amount of research has investigated human discrimination of force magnitude and force direction (Jones, 1989; Pang et al., 1991; Vicentini et al., 2010). There are different experimental paradigms such as matching tests and two alternative forced-choice discrimination tasks. Tan et al. (1994) summarised the findings from different studies (e.g., Pang et al. (1991) investigated the range: 2.5–10 kg⋅m/s^2^, Jones (1989) the range: 25–410 kg⋅m/s^2^), and reported that over a large range humans seem to exhibit a just-noticeable difference (JND) of 7% in force sensing. However, these data were measured in static, or point-based, discrimination tasks. In the current study, the force range was from 0 to 30 kg⋅m/s^2^, and was within the range reported above. Although many studies have investigated force perception, to our knowledge, there are few studies examining whether humans could understand the dynamics itself via active movements, evaluating it by static properties. The current study had a limitation but could show the transform from the active to static.

In our experiment, the visual deprivation in the test trials was introduced in order to examine force perception in isolation; however, at the same time this might have disturbed the task performance. In further examination of visual deprivation effect (see Supplementary Material 1), the nine participants showed relatively large errors in the M values, indicating they were highly influenced by the deprivation; they could be considered as visual dominant people. In addition, human decision-making process (Wolpert and Landy, 2012; Cos, 2017) is important in motor planning and force perception, especially in uncertain environments (Sober and Sabes, 2005; Sarlegna and Sainburg, 2009; Wong and Haith, 2017). We observed that most participants over-estimated their anticipated target relative to the veridical values in the test trials. This could be explained by the effect of the deprivation of visual feedback; that is, such uncertainties might have increased the delay of decision making. The over-estimation might have been caused by minimising the risk of not reaching the target. This could be also explained by a statistical decision theory such as Bayesian model (Trommershäuser et al., 2005; Nagengast et al., 2011). Besides, the current study examined human predictions of force in the range previously experienced (i.e., the half of the target). Suppose, however, participants were asked to make a prediction outside the range (2 times of the target force for example), then uncertainty would much increase, so that different brain mechanisms might be involved in anticipation and the performance change. Future experiments could test such a possibility.

Our results showed that position estimation was overall better than the force prediction. Interestingly, the direction of visual feedback synchronised with hand movements affected the performance. Comparing Figures 4B, 5B, we observe better position estimation for the vertical direction (Experiment 1) than for the radial direction (Experiment 2). In contrast, comparing Figures 4C, 5C, we see that force estimation was better in radial feedback compared with vertical. The isometric measurements, without hand movements (conducted in Supplementary Material 2), showed that both full-force and half-force estimations were relatively less accurate than other conditions with hand movements, excluding the half-force prediction in logarithmic condition. The vertical hand movements were closely related to body perception and could have had referred to the centre of body in making a position estimation. Previous research also demonstrated that postural adjustment is an important factor in motor control (Peterka, 2002; Peterka and Loughlin, 2004; Rueckert et al., 2016) and in force perception (van Beek et al., 2013).

Finally, our findings could contribute to practical applications in interactive environments. Exoskeleton technologies, for example, are increasingly used in rehabilitation and industrial fields, and it is essential to incorporate biomechanical feedback of human movements. Relevant to the present study is the need, when touching uncertain compliant surfaces, to prevent a dissociation between human force perception and impedance control, for safety reasons (Roveda et al., 2019). Further experiments would provide an optimal solution to predict and guide human motor behaviour by changing stiffness online in human-robot interaction. Moreover, an understanding of how humans maintain stable postures when interacting with external force, and an understanding of how humans predict the forthcoming state, would be valuable in developing control algorithm for self-regulating robots. Later technology might achieve a fully autonomous robot exhibiting human-like behaviour when sitting on a gym ball.

## Summary Statement

This study showed substantial differences in human estimations of force according to the stiffness profile of the environment, even though the differences in overall movement accuracy were diminished from the early stage of motor learning.

## Data Availability Statement

The original contributions presented in the study are included in the article/Supplementary Material, further inquiries can be directed to the corresponding author/s.

## Ethics Statement

The studies involving human participants were reviewed and approved by the Research Committee University of Birmingham. The patients/participants provided their written informed consent to participate in this study.

## Author Contributions

MM and JB conceived the research question. CT, MA, VR, JB, and MM developed the idea and designed the experiments. CT and VR conducted the experiments. CT, VR, MA, and JB analysed the results. CT, VR, and MA wrote the manuscript. All authors edited the manuscript.

## Conflict of Interest

The authors declare that the research was conducted in the absence of any commercial or financial relationships that could be construed as a potential conflict of interest.

## Publisher’s Note

All claims expressed in this article are solely those of the authors and do not necessarily represent those of their affiliated organizations, or those of the publisher, the editors and the reviewers. Any product that may be evaluated in this article, or claim that may be made by its manufacturer, is not guaranteed or endorsed by the publisher.
