# Supplementary material for: Human Stiffness Perception and Learning in Interacting With Compliant Environments
Source: Front Neurosci. 2022 Jun 6;16:841901. doi: 10.3389/fnins.2022.841901 (PMC9215212; doi:10.3389/fnins.2022.841901)
Supplement: Supplementary file 1 [file Data_Sheet_1.docx]

***Supplementary information***

**1. Effects of deprivation of visual feedback**

The constant overshoots from the target were mostly observed across all the participants in the reaching movements, in particular in the test trials where no visual target information was provided. Here, further examination was conducted on learning performance by analysing position accuracies when participants estimated *full-force* in trials in the learning block.

In contrast to the learning metric evaluation (Fig. 3 in the main text), no typical learning-curves were observed through the 10 test trials for all the three stiffness profiles (See Fig. S1-A). Interestingly, the graph showed that the "quadratic" case was slightly better in M values than other two cases, and this was a similar trend with Fig. 3. Because this study was designed to examine whether a participant properly learned the stiffness profile, each block consisted of a certain number of repetitions with visual information of the target to learn, followed by a trial with no visual information. However, such experimental design might have produced undesirable disturbance on their performance caused by the sudden deprivation of visual information in the trial, and also the degree of influence might have differed on an individual basis. Therefore, to evaluate the visual deprivation effect, we computed the metrics values: *M_n_*, so-called *normalised M* values, where the metric in the trial: *M_test_* was divided by the sum of the learning metric values: *M_l_* in the repetitive period prior to the trial:

$M_{n}=\frac{M_{test}}{\sum_{i=1}^{5} M_{l}(i)}$ (S1)

In comparison between Fig. S1-A and S1-B, even though the latter was normalised by the prior performance with visual information, the trends were similar between the two figures, indicating that the deprivation of visual feedback in the trial seemed to be affected with a similar degree through the entire block. Noticeably, some of the participants performed higher overshoots in the trials. Hence, the participants were divided into two groups based on *M_test_* values: low-error group (<12 ± 2 cm) and high-error group (>12 ± 2 cm). A total of 9 participants out of 36 were identified as high-error group and their normalised performance is depicted in Fig. S1-C. These figures indicate a large contribution of the high-error group onto the trend of the mean performances. The participants in the high-error group performed large overshoots from the actual goal in the trials and showed relatively large metric values in the overall performances. Such group difference might have been largely affected by a degree of their speed control accuracy in reaching movements.

Compared between the two groups, the high-error group (n = 9) showed no significant differences on metric values among three stiffness profiles (*M_n_* = 4.268, *SD* = 3.960 for "linear", *M_n_* = 1.570, *SD* = 1.035 for "quadratic", *M_n_* = 3.852, *SD* = 3.712 for "logarithmic"), *F*(2,52) = 1.710, *p* = .212. These subjects seemed to be visual dominant people in motor control and very sensitive to the deprivations of visual feedback. In contrast, the low-error group (n = 27) showed significant differences among profiles (*M_n_* = 1.144, *SD* = 0.576 for "linear", *M_n_* = 0.825, *SD* = 0.432 for "quadratic", *M_n_* = 1.457, *SD* = 0.916 for "logarithmic"), *F*(2,52) = 6.292, *p* = .004. The low-error group performed differently in the three stiffness profiles; "quadratic" was better than "linear" (*t*(1,26) = 2.823, *p* = .009) and also "logarithmic" (*t*(1,26) = 3.182, *p* = .004). Such examinations above would be helpful in evaluating whether the participants relied on visual information in reaching movements and/or whether the overshoots from the actual target was caused by the stiffness profile itself (See Discussion in the main text).

**2. Isometric force measurements**

To examine the half-estimation performance without hand movements, we conducted isometric force measurements. The higher stiffness (k = 1000 kg/s^2^) was set at z = 0 position to prevent any movements when applying force. The visual feedback was provided for a radial direction representing the force magnitude in the same way described in Experiment 2. All the experimental procedure and the protocol were similar to the main experiment, but this additional experiment consisted of two sessions only: full-force prediction and half-force prediction. The total experimental time was approximately 15minutes. Out of 14 participants in Experiment 2, ten participants (1 female, 23-31 years old, mean age: 25.50 ± 2.42 (SD)) took part in this test.

Fig. S2 shows the estimation differences between the isometric measurements and Experiment 2. A paired-samples t-test was conducted and compared the estimations between the conditions. In the full-force estimations, the estimation in the linear case was statistically better than the isometric case: *t*(9) = 2.351, *p* = .043, and the estimation in the logarithmic case was also better: *t*(9)= 2.292, *p* = .048. In the half-force estimations, the estimation in the quadratic case was significantly better than the isometric case: *t*(9) = 3.283, *p* = .009. Interestingly, the estimation in logarithmic case was significantly worse than the isometric case: *t*(9) = 2.314, *p* = .047. Excluding this logarithmic case, relatively better estimations were observed along with hand movements.

**3. Subjective stiffness perception in relation to everyday activities: Questionnaire survey**

The force perception is very subjective, and the estimations could have been largely influenced by individual experiences. After each session, we conducted a questionnaire to examine whether participants could associate the experimental stiffness profiles to the force they have experienced in everyday activities. The questionnaire consisted of five categories: 1) pushing a mattress/cushion, 2) pushing a revolving door, 3) pushing a box on a plane surface, 4) pushing a hinged door, 5) pushing a box on an inclined surface. Participants were asked to choose the most similar one to the force they experienced in the experiment.

The results are shown in Fig. S3. In the first group (n = 36) who completed the Experiment 1 where visual feedback was vertically provided in the same direction as hand movements, there was less noticeable trend among three force conditions. In contrast to the first group, there was noticeable differences among the conditions in the second group (n = 14) who completed the Experiment 2 where visual feedback was provided to the radial direction. According to the equi-probability model, a participant would respond to each Qs equally when they do not differentiate the categories; that is, 20 percent each in our questionnaire. In the first group, according to non-parametric Chi-square test, there were significant differences in their responses in the linear case (*x^2^*(4) = 17.899, *p* = .001), but no significant in quadratic (*x^2^*(4) = 5.389, *p* = .250) and in logarithmic (*x^2^*(4) = 3.722, *p* = .445). The participants might have been confused by the direction differences between hand movements in the experiment and the everyday activities asked in the questionnaire. In the second group where visual feedback was independent of the hand movements, their response trend related to the stiffness profiles can be seen in Fig. S3. Interestingly, most participants answered "pushing a revolving door" in the logarithmic stiffness condition. Although the sample size was insufficient (n = 14) to do further statistical evaluations, the results suggest a potential experiment to examine whether humans could associate such experimental stiffness profiles to their experience.


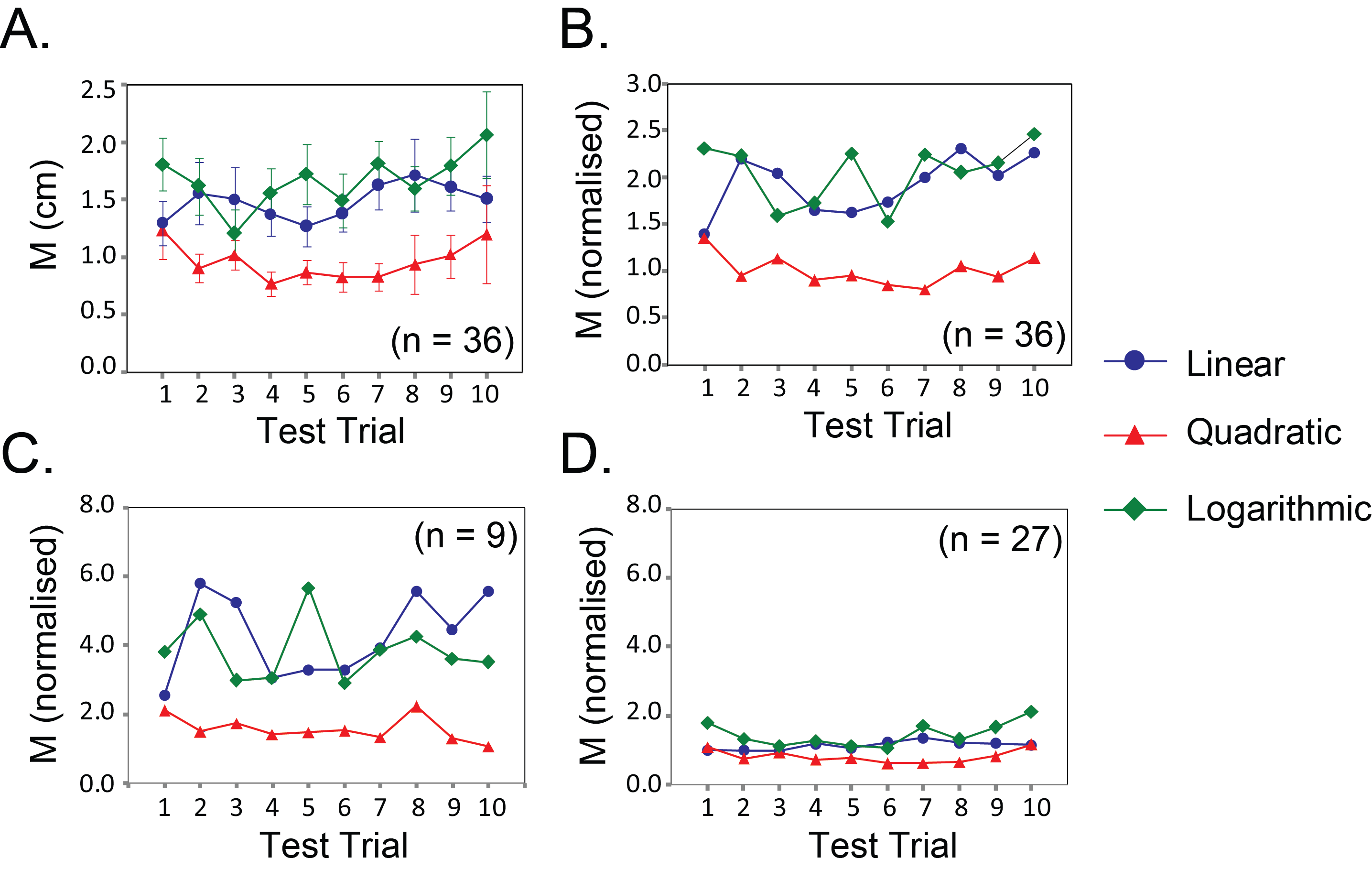


**Figure S1.** Metric values and the normalized ones, over the course of 10 test trials in the learning block. **A.** M values averaged across 36 participants with the error bars representing ± 1 standard errors. **B.** Mn values (n = 36). **C.** Mn values in the high error group (n = 9). **D.** Mn values in the low error group (n = 27).


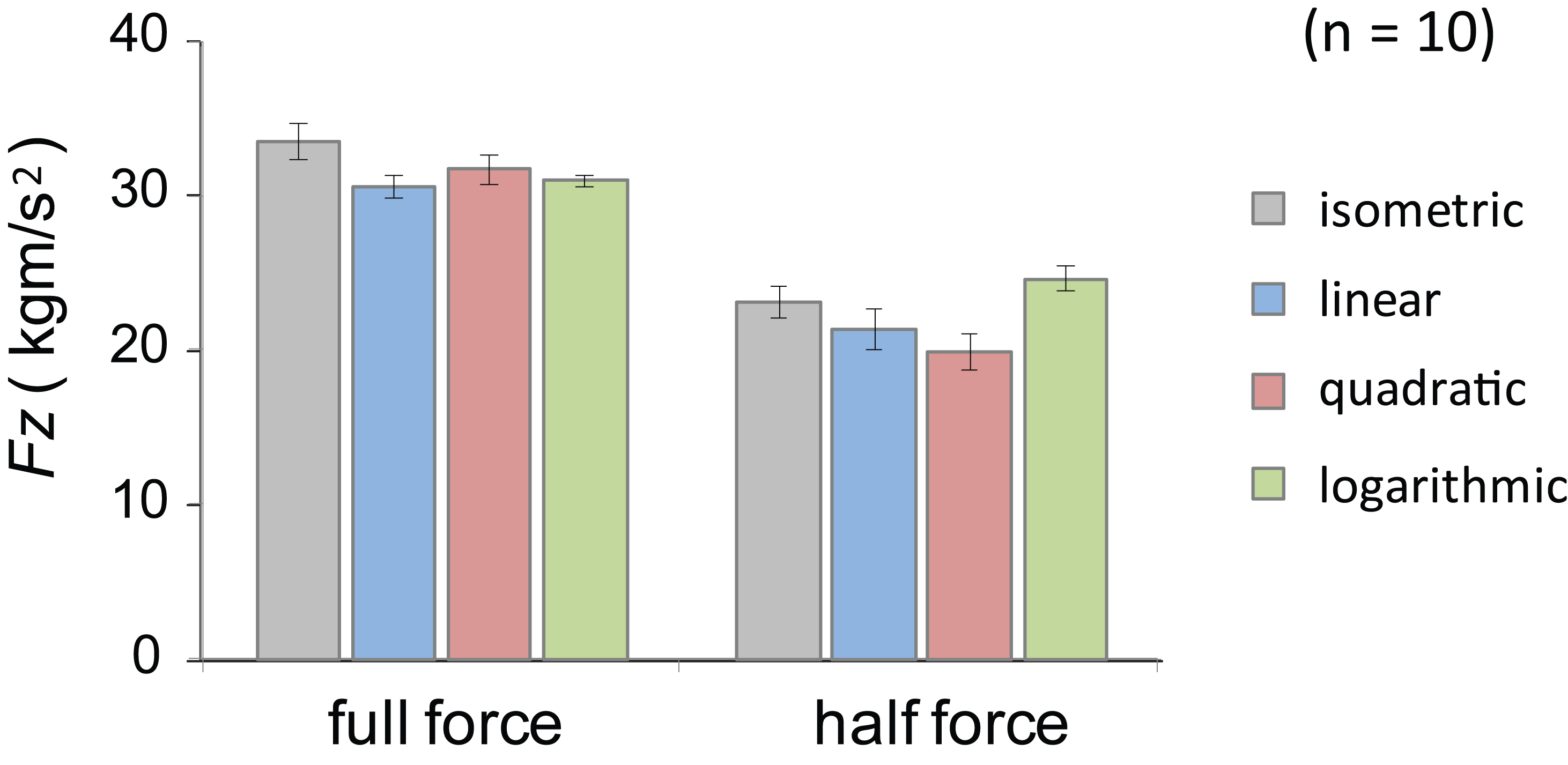


**Figure S2.** Averaged performance across 10 participants, compared between the isometric measurement (grey colour bars) and three stiffness conditions (coloured-bars) at the full-force and the half-force test trials. Error bars represent ± 1 standard errors.


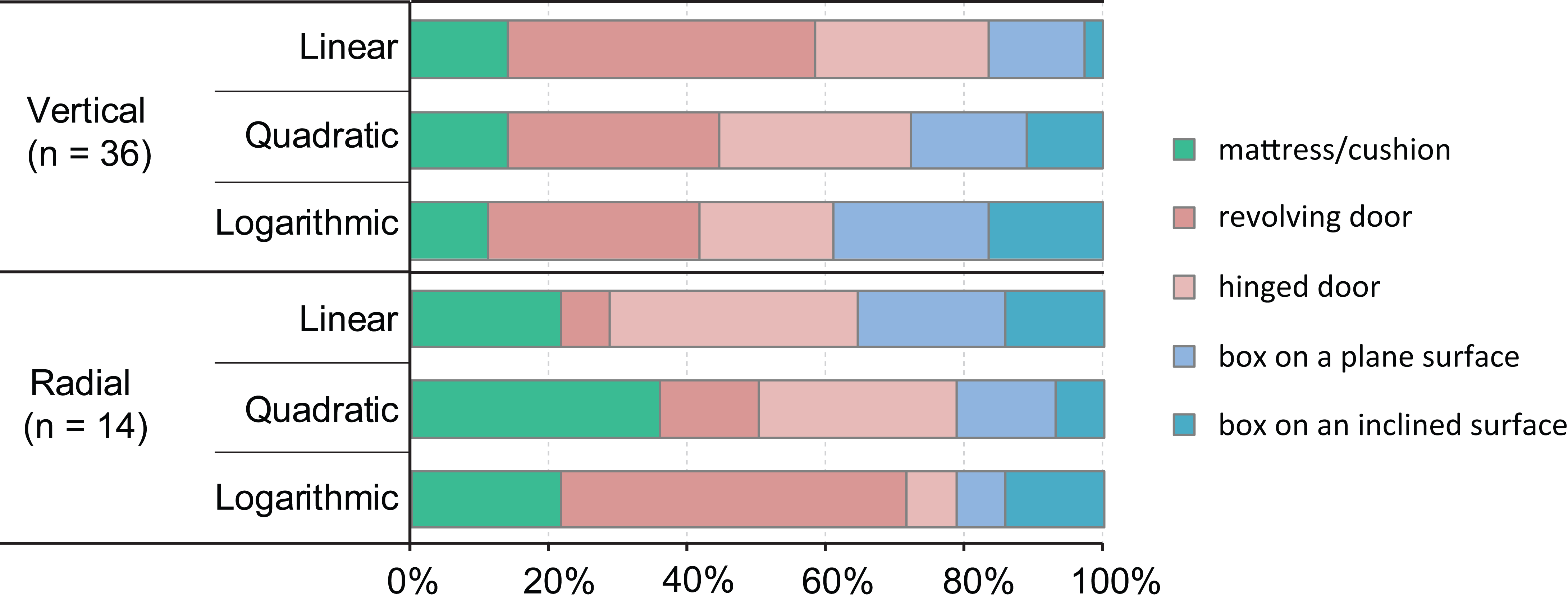


**Figure S3.** Comparisons in percentage of the number of answers in 5 items after each session which was assigned one of stiffness conditions, between the first group (36 participants in Experiment 1) and the second group (14 participants in Experiment 2).
